# Associations of neurodevelopmental traits and adverse childhood experiences with problem gambling: A nationwide cross‐sectional study

**DOI:** 10.1002/pcn5.70378

**Published:** 2026-08-05

**Authors:** Kazusa Miyahara, Atsuko Nagaoka, Mizuki Hino, Akiko Sato, Yuhei Mori, Risa Shishido, Masataka Hatano, Yuto Hosogai, Takahiro Tabuchi, Hiroaki Tomita, Yasuto Kunii

**Affiliations:** ^1^ Department of Disaster Psychiatry International Research Institute of Disaster Science Tohoku University Sendai Miyagi Japan; ^2^ Department of Neuropsychiatry, School of Medicine Fukushima Medical University Fukushima Fukushima Japan; ^3^ Department of Psychiatry Tohoku University Hospital Sendai Miyagi Japan; ^4^ Division of Epidemiology, School of Public Health Tohoku University Graduate School of Medicine Sendai Miyagi Japan; ^5^ Department of Psychiatry, Graduate School of Medicine Tohoku University Sendai Miyagi Japan

**Keywords:** adverse childhood experiences, attention‐deficit/hyperactivity disorder, autism spectrum disorder, gambling, problem gambling

## Abstract

**Aim:**

Attention‐deficit/hyperactivity disorder (ADHD) is a recognized related factor for gambling problems. Although ADHD frequently co‐occurs with autism spectrum disorder (ASD) and adverse childhood experiences (ACEs), the interaction of these factors on gambling has not been fully explored in a large general population sample. We aimed to investigate the associations of ADHD traits, ASD traits, and ACEs with problem gambling.

**Methods:**

Using data from an internet survey that includes 32,000 Japanese participants, we evaluated the associations of ADHD traits, ASD traits, ACEs scores, and their interaction with problem gambling ratio through multivariable logistic regression analysis controlling for 14 confounders. We used the Problem Gambling Severity Index to assess problem gambling, the Japanese version of the Adult ADHD Self‐Report Scale (ASRS) to assess ADHD traits, the ten‐item Japanese version of the Autism‐Spectrum Quotient (AQ‐10) to assess ASD traits, and the ten‐item Japanese version of the questionnaire to assess ACEs.

**Results:**

Problem gamblers exhibited significantly higher weighted rates of ASRS positive, AQ‐10 positive, and high ACEs score. The weighted multivariable logistic regression analysis showed that ASRS positive, AQ‐10 positive, and high ACEs score were significantly associated with problem gambling. Additionally, a significant interaction between ASRS positive and AQ‐10 positive was observed.

**Conclusion:**

ADHD traits, ASD traits, and high ACEs scores were associated with higher odds of problem gambling. The co‐occurrence of ADHD traits and ASD traits showed a negative multiplicative interaction on the odds ratio of problem gambling.

## INTRODUCTION

Problem gambling is one of the most harmful behavioral addictions, affecting not only individuals who gamble but also broader social and economic systems worldwide,^1^, ^2^ posing a significant public health concern.^3^ Its adverse consequences include relationship conflicts, criminal behavior, emotional distress,^1^ and substantial financial burdens.^2^ The prevalence of problem gambling varies globally; a previous systematic review reported rates ranging from 5.8% in the highest prevalence country to 0.12% in the lowest.^4^ Despite its serious impact and relatively high prevalence in some regions, it is estimated that only 0.23% of individuals with problem gambling seek professional help.^5^ Moreover, current strategies for early detection and intervention have shown only limited effectiveness. Therefore, identifying comprehensive related factors and developing effective screening systems for high‐risk groups are essential to prevent the onset and progression of problem gambling.

Attention‐deficit/hyperactivity disorder (ADHD) is a neurodevelopmental condition characterized by catecholaminergic dysfunction, particularly involving the dopamine and noradrenaline systems,^6^ and is recognized as a related factor for problem gambling. A meta‐analysis reported that 9.3% of help‐seeking individuals with problem gambling had comorbid ADHD.^7^ Notably, even when ADHD symptoms do not meet the diagnostic threshold, subclinical ADHD traits have been identified as related factors for problem gambling. A large‐scale study involving over 7000 individuals in the United Kingdom found that those with ADHD symptoms had a higher prevalence of problem gambling than those without such symptoms.^8^ Additionally, comorbid ADHD has been shown to negatively impact clinical outcomes in individuals with gambling disorders, with co‐occurring cases experiencing more frequent and severe relapses.^9^ Collectively, these findings suggest that ADHD contributes not only to the development of problem gambling but also to its clinical course and prognosis.

Autism spectrum disorder (ASD) and adverse childhood experiences (ACEs) are also associated with ADHD; however, their relationships with problem gambling remain insufficiently explored. ASD is a neurodevelopmental disorder that shares molecular and genetic features with ADHD,^10^ and the two conditions frequently co‐occur, with approximately 13% of children diagnosed with ADHD also meeting criteria for ASD.^11^ However, it remains unclear whether ASD is an independent related factor for problem gambling. A systematic review of 30 studies reported a positive association between ASD and behavioral addictions.^12^ However, as to association with gambling, it involves only one study, leaving gambling‐specific evidence limited. In contrast, other research suggests that ASD traits are significantly associated with lower rates of addiction.^13^, ^14^ These inconsistencies may stem from interaction with ADHD. A previous study involving 197 Japanese participants reported that while ASD traits alone were negatively associated with problem gambling, the co‐occurrence of ADHD and ASD traits was positively associated.^13^ Given the small sample size of that study, further research in a larger cohort is warranted to clarify these associations.

Children with ADHD and/or ASD are more frequently exposed to ACEs than are children with typical development.^15^, ^16^ ACEs have been recognized as significant contributors to adverse behavioral health outcomes, including suicidal behaviors,^17^ tobacco use,^18^ and Internet addiction, such as online gambling.^19^ Although the association between ACEs and problem gambling has been well‐documented,^20^, ^21^ the specific relationship of ACEs to problem gambling among individuals with ADHD or ASD traits remains unclear.

While ADHD, ASD, and ACEs are all critical factors in the development of problem gambling, their joint associations have not been examined in large, population‐based cohorts. Therefore, we aimed to investigate the independent and joint associations of ADHD traits, ASD traits, and ACEs with gambling behavior.

## METHODS

### Design and participants

This study utilized data from the 2024 wave of the Japan Society and New Tobacco Internet Survey (JASTIS) study,^22^ conducted by Rakuten Insight, Inc. (Tokyo, Japan) between January 24 and February 27, 2024. The detailed study design and protocol have been previously described.^23^, ^24^ Briefly, the survey targeted a nationally representative sample of 32,000 participants, reflecting a broad range of demographic and socioeconomic backgrounds. Of the 32,000 respondents, 2732 were excluded based on the following criteria: extremely short response time (less than 600 s), failure to answer an attention‐check question, endorsement of all listed drug use categories or all listed health conditions or reporting more than 15 family members. After these exclusions, 29,268 participants were included in the final analysis.

### Problem gambling

Participants were asked whether they had engaged in any of the following gambling activities: offline or online horse racing, bicycle racing, boat racing, or auto racing; offline or online casinos; offline or online lotteries; offline or online sports promotion lotteries; stock, commodities, or foreign exchange trading; pachinko/pachinko slot machines; and cryptocurrency trading. Based on their responses, participants were categorized into three levels of gambling involvement: never (selected “never” for all types of gambling), current (selected “within a year [less than weekly]” or “within a year [at least weekly]” for any gambling activity), and past (selected other responses indicating previous experience without current use).^24^ Problem gambling was assessed using the Problem Gambling Severity Index (PGSI),^25^ a validated 9‐item instrument commonly used to identify problem gambling, which has been validated in the Japanese population.^26^ According to the PGSI scoring system, a total score of 0 indicates no gambling problems, scores of 1–2 indicate low risk, 3–7 indicate moderate risk, and 8–27 indicate problem gambling.^25^

### ADHD screening

ADHD traits were assessed using the 6‐item Japanese version of the Adult ADHD Self‐Report Scale (ASRS‐J‐6).^27^ Participants were classified as ASRS positive if they endorsed four or more items above the clinical threshold, consistent with established screening criteria.

### ASD screening

ASD traits were evaluated using the 10‐item Japanese version of the Autism‐Spectrum Quotient (AQ‐10), a brief self‐administered screening tool derived from the full AQ.^28^ In this study, participants were considered AQ‐10 positive if they scored above the threshold on 7 or more of the 10 items.

### ACEs scoring

ACEs exposure was assessed using the 10‐item Japanese version of a questionnaire commonly employed in previous ACEs studies.^29^, ^30^ Participants were asked about exposure to adverse experiences such as psychological and physical abuse before the age of 18 years. Each item was scored as 0 (no) or 1 (yes), yielding a total score ranging from 0 to 10, representing the cumulative number of ACEs. In this study, participants were considered high ACEs score if they scored above the threshold on four or more of the 10 items as with the previous study.^29^

### Covariates

To examine the associations between ADHD traits, ASD traits, ACEs, and problem gambling, we included demographic, socioeconomic, occupational, and health‐related variables as covariates in the multivariable model, following a previous study.^24^ Demographic, socioeconomic, and health‐related variables were selected based on a prior meta‐analysis of related factors for problem gambling,^31^ while occupational variables were included based on evidence from earlier studies demonstrating potential associations with problem gambling.^32^, ^33^ Demographic and socioeconomic variables included age group (ranging from ≤24 years to 75–82 years), sex (man or woman), and marital status (married, never married, or widowed/separated). Socioeconomic status was further assessed by educational attainment (high school or less vs. college or higher) and annual household income, categorized as follows: ≤4.0 million JPY, 4.1–6.0 million JPY, 6.1–8.0 million JPY, 8.1–10.0 million JPY, 10.1–12.0 million JPY, 12.1–15.0 million JPY, and ≥15.1 million JPY. Occupational variables included employment status (employer, self‐employed, regular employee, or part‐time employee). Participants' working industries were classified into the following categories: agriculture, forestry, fishing, mining, utilities, construction, manufacturing, wholesale trade, retail trade, transportation, information, finance, insurance, real estate, education, healthcare, social assistance, accommodation, food services, and other services.

Health‐related variables included smoking status (never, past, or current); alcohol consumption assessed using the Alcohol Use Disorder Identification Test, categorized as no or low‐risk use (0–7 points), medium‐risk use (8–15 points), high‐risk use (16–19 points), and likely alcohol dependence (20–40 points)^34^; mental health status assessed using the Kessler‐6 scale (no psychological distress, 0–4 points; moderate distress, 5–12 points; and serious distress, 13–24 points)^35^; and coexisting psychiatric conditions, defined as the presence or absence of depression and other psychiatric disorders.

### Statistical analyses

We employed the inverse probability weighting (IPW) method to adjust for differences between internet survey respondents and the general population, as previously described.^8^, ^36^ The reference population was derived from the 2019 Comprehensive Survey of Living Conditions (CSLC) conducted by the Ministry of Health, Labour and Welfare. Briefly, data from the two surveys were merged and stratified by sex and age group (14 strata), and propensity scores were estimated using multivariable logistic regression for each stratum. In most strata, adjustments were made for variables, including residential area, marital status, educational attainment, household homeownership, self‐rated health, and smoking status.

To analyze sample characteristics, we used the chi‐square test for categorical variables and Student's *t*‐test for continuous variables. Weighted Spearman's rank correlation coefficients were calculated to assess the associations between ASRS and high ACEs scores and between AQ‐10 and high ACEs scores. Multivariable logistic regression models were used to examine the associations between problem gambling and ASRS, AQ‐10, and high ACEs scores, adjusting for the aforementioned covariates (sex, age, employment, industry, worktime, marital state, psychological distress, alcohol use, cigarette and tabacco use, depression, psychiatric disorder, educational attainment, and income). We set ASRS positive, AQ‐10 positive, and high ACEs scores as target associations, while others as adjustment variables. We calculated adjusted predicted probabilities of problem gambling with 95% confidence interval (CI) based on this model. Additionally, we also conducted sensitivity analysis using continuous ASRS, AQ‐10, and ACE scores and setting the same adjustment variables to avoid the effect of dichotomizing continuous scales.^37^

Model fit was assessed using the Hosmer–Lemeshow (HL) goodness‐of‐fit test. Statistical significance was set at *p* < 0.05 for all analyses, except for the HL test, for which *p* > 0.05 was considered indicative of good model fit. All analyses were performed using R version 4.4.2.

### Ethical considerations

Participants provided online informed consent prior to completing the survey. As compensation, participants received monetary points redeemable for online purchases. All study procedures were conducted in accordance with the Declaration of Helsinki and the Ethical Guidelines for Medical and Health Research Involving Human Subjects. The study protocol was approved by the ethics committee of Tohoku University Graduate School of Medicine.

## RESULTS

**Figure 1:**
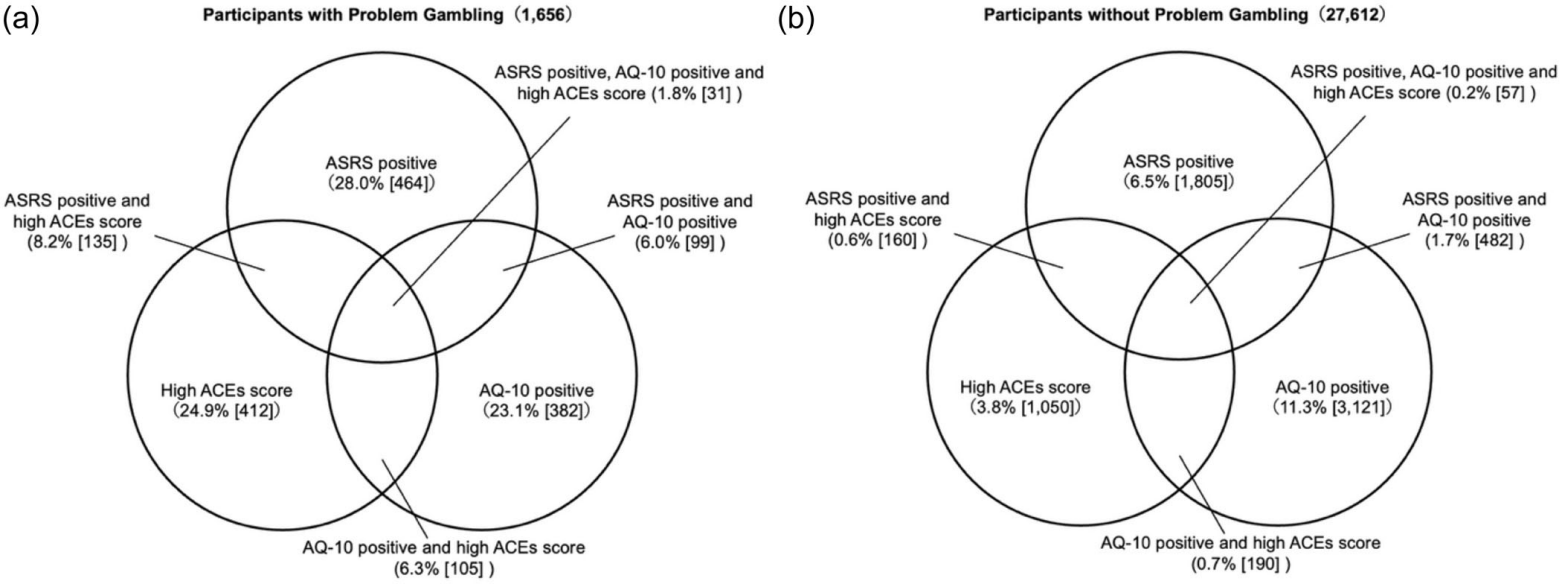
Proportions of Adult attention‐deficit/hyperactivity disorder Self‐Report Scale (ASRS) positive, ten‐item Japanese version of the Autism‐Spectrum Quotient (AQ‐10) positive, and high adverse childhood experiences (ACEs) score. Venn diagrams showing proportions of ASRS positive, AQ‐10 positive, and high ACEs score of (a) participants with problem gambling (Problem Gambling Severity Index [PGSI] is eight or more) and (b) participants without problem gambling (PGSI is seven or less). Each number represents the proportion (%) [the number of participants].

**Figure 2:**
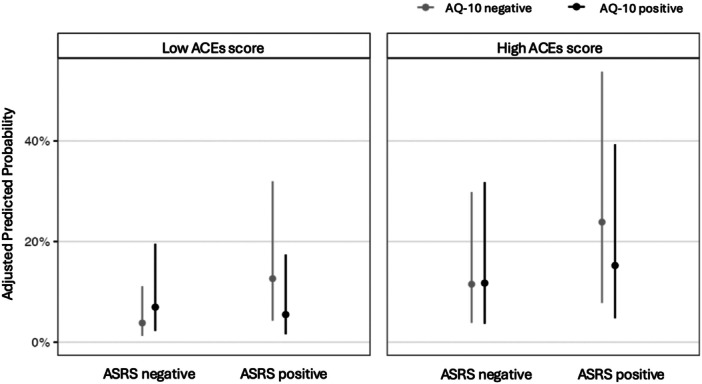
Interaction plot showing adjusted predicted probability of problem gambling with 95% confidence interval. ACEs, adverse childhood experiences; AQ‐10, ten‐item Japanese version of the Autism‐Spectrum Quotient; ASRS, Adult attention‐deficit/hyperactivity disorder Self‐Report Scale.

Demographic characteristics of the 29,268 valid respondents eligible for inclusion in the analyses are shown in Table 1. The proportions of ASRS positive, AQ‐10 positive, and high ACEs score are provided in Figure 1. Weighted correlation coefficient between ASRS and high ACEs score was 0.151, while that between AQ‐10 and high ACEs score was 0.066. For gambling status, “current” gamblers, meaning gambling at the time of the survey, exhibited significantly higher weighted ASRS positive rate, AQ‐10 positive rate, and high ACEs score than others (ASRS positive rate, 7.5% vs. 6.6%, *p* < 0.001; AQ‐10 positive rate, 12.9% vs. 11.6%, *p* < 0.001; and high ACEs score, 6.9% vs. 3.8%, *p* < 0.001) (Supporting Information S1: Figure 1). Similarly, problem gamblers exhibited significantly higher weighted rates of ASRS positive, AQ‐10 positive, and high ACEs score (ASRS, 27.3% vs. 5.8%, *p* < 0.001; AQ‐10, 24.0% vs. 11.4%, *p* < 0.001; and ACEs, 22.8％ vs. 4.0%, *p* < 0.001) (Supporting Information S1: Figure 2).

**Table 1:** Demographic data of study participants with and without problem gambling.

	Participants without problem gambling	Participants with problem gambling	*p*‐Value
Number of participants	27,612	1656	
Age: mean (SD)	49.5 ± 17.5	36.1 ± 12.9	<0.001
*Sex*			<0.001
Male: *n* (%)	13,208 (47.8%)	1244 (75.1%)	
Female: *n* (%)	14,404 (52.2%)	412 (24.9%)	
*ASRS*			<0.001
Positive: *n* (%)	1805 (6.5%)	464 (28.0%)	
Negative: *n* (%)	25,807 (93.5%)	1192 (72.0%)	
*AQ‐10*			<0.001
Positive: *n* (%)	3121 (11.3%)	382 (23.1%)	
Negative: *n* (%)	24,491 (88.7%)	1274 (76.9%)	
*ACEs score*			<0.001
Low ACEs score: *n* (%)	26,562 (96.2%)	1244 (75.1%)	
High ACEs score: *n* (%)	1050 (3.80%)	412 (24.9%)	

Abbreviations: ACEs, adverse childhood experiences; AQ‐10, ten‐item Japanese version of the Autism‐Spectrum Quotient; ASRS, Adult attention‐deficit/hyperactivity disorder Self‐Report Scale; SD, standard deviation.

Next, to investigate the associations of ADHD traits, ASD traits, and ACEs with problem gambling adjusting confounders, we conducted multivariable logistic regression analyses. When other variables were at their reference levels, the multivariable logistic regression model showed that problem gambling was significantly associated with ASRS positive (odds ratio [OR] 3.64, 95% CI 2.60–5.10, *p* < 0.001), AQ‐10 positive (OR 1.89, 95% CI 1.32–2.69, *p* < 0.001), and high ACEs score (OR 3.28, 95% CI 2.24–4.81, *p* < 0.001) (Table 2). In addition, we found a negative interaction between ASRS positive and AQ‐10 positive (OR 0.21, 95% CI 0.09–0.48, *p* < 0.001), while no significant interaction was observed between high ACEs score and ASRS positive (*p* = 0.30) and AQ‐10 positive (*p* = 0.10). The HL test was *p* = 0.85, indicating the logistic model fit well. Based on this model, we calculated the adjusted predicted probability of problem gambling stratified by ASRS, AQ‐10, and ACEs score. Those with ASRS positive, AQ‐10 negative, and high ACEs score exhibited the highest (Figure 2; adjusted predicted probability 0.24, 95% CI 0.08–0.54). Supporting Information S2: Table 1 presents detailed data for each combination of ASRS, AQ‐10, and ACEs statuses, including the number of participants, weighted percentage, and adjusted predicted probabilities with 95% CIs of problem gamblers.

**Table 2:** Results of a multivariable logistic regression analysis.

	OR	*p*‐Value	95% CI
Intercept	0.04	<0.001	0.01, 0.13
*ASRS* a			
Negative	Reference		
Positive	3.64	<0.001	2.60, 5.10
*AQ* a			
Negative	Reference		
Positive	1.89	<0.001	1.32, 2.69
*ACEs* a		<0.001	
Low ACEs score	Reference		
High ACEs score	3.28	<0.001	2.24, 4.81
*Sex*			
Male	Reference		
Female	0.38	<0.001	0.28, 0.51
*Age*			
15–24	Reference		
25–34	0.68	0.08	0.45, 1.04
35–44	0.30	<0.001	0.19, 0.48
45–54	0.20	<0.001	0.12, 0.34
55–64	0.19	<0.001	0.11, 0.33
65 or more	0.17	<0.001	0.08, 0.37
*Employment*			
Employer	Reference		
Part‐time employee	0.42	<0.001	0.26, 0.67
Regular employee	0.48	<0.001	0.33, 0.70
Self‐employed	0.48	0.008	0.28, 0.83
*Industry*			
Agriculture, forestry, fishing, mining	Reference		
Accommodation and food services	2.33	0.17	0.70, 7.72
Construction	1.95	0.22	0.67, 5.69
Education services	2.00	0.18	0.72, 5.57
Finance, insurance	2.65	0.07	0.92, 7.66
Healthcare	1.89	0.23	0.66, 5.40
Information services	1.64	0.35	0.58, 4.64
Manufacturing	2.23	0.12	0.82, 6.07
Wholesale trade	2.01	0.20	0.68, 5.91
Real estate	1.51	0.51	0.44, 5.16
Retail trade	1.65	0.35	0.57, 4.73
Transportation	2.08	0.18	0.71, 6.07
Utilities	2.08	0.25	0.60, 7.15
Welfare	1.93	0.26	0.61, 6.12
Other services	1.70	0.30	0.63, 4.58
*Worktime*			
Non‐night‐shift work	Reference		
Night‐shift work	1.31	0.05	1.00, 1.71
*Marital status*			
Married	Reference		
Never married	0.78	0.06	0.60, 1.01
Widowed/separated	0.97	0.89	0.62, 1.51
*Psychological distress measured by the Kessler‐6 scale*			
No (0–4 points)	Reference		
Moderate (5–12 points)	3.56	<0.001	2.71, 4.68
Serious (13–24 points)	3.78	<0.001	2.71, 5.28
*Alcohol use measured by the AUDIT*			
Low‐risk use	Reference		
Medium‐risk use	0.51	<0.001	0.38, 0.69
High‐risk use	1.42	0.09	0.95, 2.13
Likely alcohol dependent	2.99	<0.001	2.19, 4.07
*Combustible cigarette use*			
Never	Reference		
Current	2.31	<0.001	1.49, 3.58
Past	1.94	<0.001	1.35, 2.80
*Heated tobacco product use*			
Never	Reference		
Current	2.58	<0.001	1.84, 3.63
Past	2.13	<0.001	1.53, 2.98
*Depression*			
Absent	Reference		
Present	1.91	<0.001	1.37, 2.64
*Psychiatric disorder*			
Absent	Reference		
Present	1.44	0.07	0.97, 2.12
*Educational attainment*			
High school graduate or less	Reference		
College graduate or more	0.87	0.19	0.71, 1.07
Other	0.31	0.10	0.07, 1.27
*Income*			
4.0 million JPY or less	Reference		
4.1–6.0 million JPY	1.12	0.46	0.82, 1.53
6.1–8.0 million JPY	1.11	0.54	0.80, 1.54
8.1–10.0 million JPY	1.15	0.48	0.79, 1.67
10.1–12.0 million JPY	0.91	0.73	0.52, 1.59
12.1–15.0 million JPY	0.83	0.66	0.37, 1.88
15.1 million JPY and more	1.07	0.82	0.61, 1.85
*Interaction term* a			
ASRS positive and high ACEs score	0.66	0.30	0.30, 1.45
ASRS positive and AQ positive	0.21	<0.001	0.09, 0.48
High ACEs score and AQ positive	0.54	0.10	0.26, 1.13
ASRS positive, high ACEs score, and AQ positive	2.64	0.17	0.66, 10.57

Abbreviations: ACEs, adverse childhood experiences; AQ‐10, ten‐item Japanese version of the Autism‐Spectrum Quotient; ASRS, Adult attention‐deficit/hyperactivity disorder Self‐Report Scale; CI, confidence interval; OR, odds ratio.

^a^ Target associations.

We also conducted sensitivity analysis using continuous ASRS, AQ‐10, and ACE scores and setting the same adjustment variables. Consistently, problem gambling was positively associated with ASRS score (OR 2.24, 95% CI 1.82–2.76, *p* < 0.001), AQ‐10 score (OR 1.46, 95% CI 1.30–1.64, *p* < 0.001), and ACEs score (OR 1.53, 95% CI 1.21–1.93, *p* < 0.001) (Supporting Information S2: Table 2). Significant interaction was also observed between ASRS and AQ‐10 score (OR 0.90, 95% CI 0.87–0.94, *p* < 0.001).

## DISCUSSION

In this study, we investigated whether neurodevelopmental traits and exposure to ACEs are associated with gambling engagement, using data from an internet‐based survey. The prevalence of problem gambling was approximately 5%, a rate consistent with previous findings in Japan.^38^ We found that ASRS and AQ‐10 positivity was a significant associated factor for problem gambling. Additionally, high ACEs scores were associated with higher odds of problem gambling. Moreover, we demonstrated a significant interaction between ASRS and AQ‐10 positivity on gambling. These findings were also consistent with the sensitivity analysis setting ASRS, AQ‐10, and ACEs scores as continuous variables. To our knowledge, this is the first study to evaluate the interplay between neurodevelopmental vulnerabilities and ACEs in relation to problem gambling in a large population‐based cohort.

The high frequency of ADHD traits among individuals with problem gambling may be explained by dysfunction of the dopaminergic reward system. ADHD has been associated with abnormalities in dopamine signaling, including genetic variations in dopamine receptors,^39^ imbalances in dopamine receptor subtypes,^40^ and altered epigenetic regulation.^41^ These dysfunctions are linked to aberrant reward system activity. For example, children with ADHD exhibit the fluctuated activity of striatum,^42^ whose increased activity has been associated with the severity of gambling behavior.^43^, ^44^ The neurobiological abnormalities in the reward system may contribute to the positive association between ADHD and problem gambling.

ASD traits were also positively associated with problem gambling in the current results. This is inconsistent with the previous study involving less than 100 valid participants, which reported the negative association between ASD traits and gambling.^13^ This inconsistency may be derived from the difference in sample size. Accounting for larger subjects involved in the current analyses, a relationship between ASD traits and high gambling odds may be plausible.

Additionally, we found the inhibitory interaction between ASRS positive and AQ‐10 positive. It is worth noting that the co‐occurrence of ASRS positive and AQ‐10 positive showed a negative multiplicative interaction on the odds ratio scale of problem gambling (OR = 0.21), even though individuals with ASRS positive and AQ‐10 positive exhibit higher odds than both negative (approximately combined OR = 1.45). This finding implies that the co‐occurrence of ADHD and ASD exhibits a distinct phenotype rather than a mere overlap of the two.^45^, ^46^ Assessing interaction terms is crucial when investigating associations of both ADHD and ASD traits with problem gambling.

High ACEs scores were also identified as an associated factor for problem gambling in the present analysis, consistent with findings of previous studies.^20^, ^21^ This may be attributable to the disruptive impact of ACEs on the development of reward circuits.^47^ ACE exposure is frequently observed in children with ADHD^15^, ^48^ and has been associated with increased severity of ADHD symptoms.^49^ However, given that the interaction between ADHD traits and ACEs was not significant in our analysis, this suggests that their co‐occurrence does not reflect a distinct neurological profile beyond that associated with either factor alone.

This study has some limitations. First, the analyzed population may have been biased. In particular, the proportion of participants who were positive on both the ASRS and the AQ‐10 was very small (approximately 2%), which may have introduced a potential bias on the interaction between the two. Although we applied IPW using data from CSLC to adjust for this, potential biases inherent to internet‐based surveys cannot be fully excluded. Second, the temporal relationship between neurodevelopmental traits and problem gambling remains unclear due to the cross‐sectional design. While we hypothesize that innate neurodevelopmental traits increase vulnerability to problem gambling, it is also possible that gambling‐related health issues may manifest as symptoms resembling ADHD or ASD. Third, neurodevelopmental traits were assessed using self‐reported screening tools (ASRS‐J‐6 and AQ‐10). Therefore, it remains uncertain whether these findings are generalizable to clinically diagnosed ADHD or ASD as defined by DSM‐5 criteria. Fourth, the specific associations of different types of ACEs were not evaluated. Prior research suggests that ACEs may differentially affect health outcomes depending on their category.^50^ Fifth, non‐linear associations of ASRS, AQ, and ACEs score were not fully evaluated in the current study. Further studies are warranted to examine the more detailed associations of those scales with problem gambling.

In conclusion, we showed that ADHD traits, ASD traits, and high ACEs scores were associated factors for problem gambling. On the other hand, comorbidities of ADHD and ASD traits have a negative interaction on problem gambling. Our findings open new avenues for understanding the connection between gambling behaviors and neuropsychiatric characteristics.

## AUTHOR CONTRIBUTIONS

Kazusa Miyahara, Mizuki Hino, and Yasuto Kunii conceived the study design. Kazusa Miyahara performed the analysis of data. Takahiro Tabuchi acquired the data. Kazusa Miyahara wrote the original draft. Other authors revised the original draft. Atsuko Nagaoka, Hiroaki Tomita, and Yasuto Kunii jointly managed the project. All the authors checked the final manuscript.

## CONFLICT OF INTEREST STATEMENT

T.T. received financial support for research (research fundings, consulting fees, or lecture fees) from Daiichi Sankyo Healthcare Co., Ltd., Johnson & Johnson K.K., Data Seed Inc., Workout‐Plus LLC, and EMMA Co., Ltd. (in the last 36 months).

## ETHICS APPROVAL STATEMENT

This study was approved by the ethics committee of Tohoku University Graduate School of Medicine and was conducted in accordance with the Declaration of Helsinki and the Ethical Guidelines for Medical and Health Research Involving Human Subjects.

## PATIENT CONSENT STATEMENT

Participants provided online informed consent prior to completing the survey.

## CLINICAL TRIAL REGISTRATION

N/A.

## Supporting information

Supporting File 1.

Supporting File 2.

## Data Availability

The data used in this study are not available in a public repository because they contain personally identifiable information. All data enquiries should be addressed to the person responsible for data management, Dr. Takahiro Tabuchi, at tabuchitak@gmail.com.
