# Supplementary material for: Associations of neurodevelopmental traits and adverse childhood experiences with problem gambling: A nationwide cross‐sectional study
Source: PCN Rep. 2026 Aug 5;5(3):e70378. doi: 10.1002/pcn5.70378 (PMC13440604; doi:10.1002/pcn5.70378)
Supplement: Supplementary file 1 [file PCN5-5-e70378-s001.docx]

**Supplementary Figure 1. Proportions of ASRS positive, AQ-10 positive, and high ACEs score by gambling engagement classification.**

Bar plots showing weighted prevalence rates of ASRS positive participants, AQ-10 positive participants, and participants with high ACEs score by gambling engagement classification. The error bar indicates 95% confidence intervals. ASRS, adult ADHD self-report scale; AQ-10, autism‐spectrum quotient; ACEs, adverse childhood experiences.

**Supplementary Figure 2. Proportions of ASRS positive, AQ-10 positive, and high ACEs score by problem gambling severity classification.**

Bar plots showing weighted prevalence rates of ASRS positive participants, AQ-10 positive participants, and participants with high ACEs score by problem gambling severity classification. The error bar indicates 95% confidence intervals. ASRS, adult ADHD self-report scale; AQ-10, autism‐spectrum quotient; ACEs, adverse childhood experiences; PGSI, problem gambling severity index.
